# GlycA measured by NMR spectroscopy is associated with disease activity and cardiovascular disease risk in chronic inflammatory diseases^☆^

**DOI:** 10.1016/j.ajpc.2020.100120

**Published:** 2020-11-07

**Authors:** Nehal N. Mehta, Amit K. Dey, Reethika Maddineni, William E. Kraus, Kim M. Huffman

**Affiliations:** a National Heart, Lung, and Blood Institute, National Institutes of Health, Bethesda, MD, 20814, USA; b Division of Cardiology, Duke Molecular Physiology Institute, Duke University School of Medicine, Durham, NC, 27701, USA; c Division of Rheumatology and Immunology, Duke Molecular Physiology Institute, Duke University School of Medicine, Durham, NC, 27701, USA

**Keywords:** GlycA, Autoimmune disease, Cardiovascular disease risk, Atherosclerosis

## Abstract

GlycA is a biomarker of systemic inflammation, quantifying both the protein concentrations and glycosylation states of several acute phase proteins. GlycA has been shown to be associated with both subclinical atherosclerosis and with cardiovascular disease (CVD). GlycA levels are higher in acute and chronic inflammation. During ongoing systemic inflammatory processes, GlycA specific acute phase reactants and proteins undergo circulating concentration and glycosylation pattern changes, and these alterations are reflected in the GlycA NMR signal. Additionally, levels associate with ongoing disease severity in individuals with rheumatoid arthritis (RA), systemic lupus erythematosus (SLE), and psoriasis thus capturing active inflammation. Furthermore, in these disease states, GlycA is associated with cardiovascular disease (CVD) independent of traditional risk factors including C-reactive protein (CRP). Finally, GlycA levels decrease with exercise, weight loss, and systemic anti-inflammatory agents. Therefore, GlycA appears to be a promising new composite biomarker of active systemic inflammation including assessing CVD risk in patients with inflammatory diseases.

## Highlights

• Patients with chronic inflammatory disorders are at increased risk for cardiovascular diseasenot captured by traditional risk factors.
• GlycA is a biomarker of acute phase reactants by NMR spectroscopy which captures disease activity in human inflammatory diseases.
• GlycA associates with cardiovascular disease and offers a tool to monitor primary disease activity and assess CVD risk in inflammatory diseases.

## 1 Introduction

**Fig. 1:**
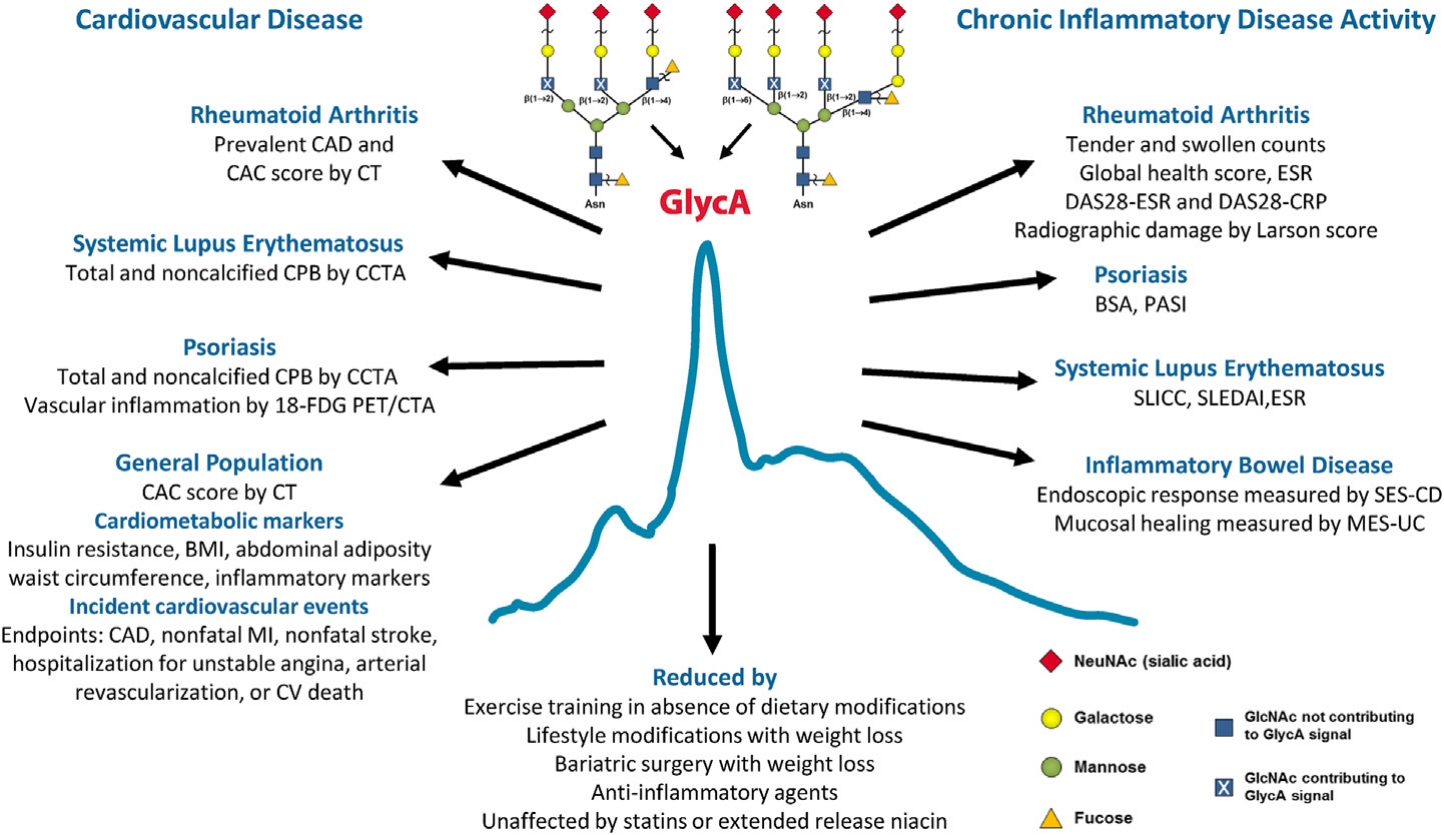
Image of the GlycA nuclear magnetic resonance (NMR) signal at approximately 2.0 ​ppm in the NMR spectrum from plasma or serum. Abbreviations: BMI, body mass index; BSA, body surface area; CAC, coronary artery calcium; CAD, coronary artery disease; CCTA, coronary computed tomographic angiography; CPB, coronary plaque burden; CRP, C-reactive protein; CT, computed tomography; CV, cardiovascular; DAS, disease activity score; ESR, erythrocyte sedimentation rate; 18-FDG PET/CTA, 18-F fluorodeoxyglucose positron emission tomography computed tomography angiography; MI, myocardial infarction; PASI, psoriasis area severity index; SLEDAI, systemic lupus erythematosus disease activity index; SLICC, systemic lupus international collaborating committee.

GlycA is a biomarker of systemic inflammation, quantifying both the protein concentrations and glycosylation states of several acute phase proteins (Fig. 1). GlycA has been shown to be associated with both subclinical atherosclerosis and with cardiovascular disease (CVD). GlycA levels are higher in acute and chronic inflammation whereby GlycA-specific acute phase reactants and proteins undergo circulating concentration and glycosylation pattern changes. These alterations are reflected in the GlycA NMR signal readily measurable with routine lab assessment. In this review, we will cover how GlycA levels associate with ongoing disease severity in individuals with rheumatoid arthritis (RA), systemic lupus erythematosus (SLE), and psoriasis as well as various forms of CVD in those with or without chronic inflammation.

### 1.1 Chronic inflammatory diseases increase the risk of cardiovascular disease development

Patients with autoimmune inflammatory disorders such as rheumatoid arthritis (RA), systemic lupus erythematosus (SLE) and psoriasis have a combined prevalence as high as 18% of the global population and are at increased risk for inflammation driven cardiovascular disease (CVD) [[1], [2], [3], [4], [5], [6], [7], [8], [9], [10], [11], [12], [13], [14]]. Inflammation drives atherosclerosis, ischemic heart disease, and heart failure; in part through atherosclerotic plaque instability [12,13,15,16]. The increased risk of CVD events in autoimmune disease has been attributed to a combination of autoimmune disease-related systemic inflammation and a greater prevalence of traditional CVD risk factors, including type 2 diabetes, physical inactivity, dyslipidemia, hypertension and obesity [13,[17], [18], [19]]. Consequently, to reduce CVD in patients with autoimmune disorders, recommendations are to minimize disease activity and actively address traditional CVD risk factors [[17], [18], [19]]. These recommendations have been incorporated into guidelines from the AHA/ACC as well as the AAD/EULAR for both RA, SLE, and, more recently, psoriasis management [20,21]. However, addressing CVD risk in chronic inflammation is complicated. In RA, CVD risk assessment and traditional lipid panels—such as low density lipoprotein cholesterol (LDL-C) are not good predictors of CVD risk [12,13,17,22,23]. The presence of seemingly normal or low LDL-C and HDL-C despite greater CVD risk is referred to as the “lipid paradox” [17,[22], [23], [24], [25]]. Furthermore, recent evidence has also demonstrated that inflammatory markers such as high sensitivity C-reactive protein (hsCRP)—may not accurately predict cardiovascular disease risk in inflammatory disease states such as systemic lupus erythematosus [26], psoriasis [27], and rheumatoid arthritis [28]. The lipid paradox and additional effects of masked inflammation reduce the accuracy of typical CVD risk estimators when used in chronic inflammatory diseases. This is important to address since the interaction between systemic inflammation and lipoprotein leads to smaller, atherogenic LDL particles with reduced high density lipoprotein (HDL)-C accelerating inflammation-associated atherosclerosis [12,17,[22], [23], [24], [25]]. While hsCRP is a reliable predictor of prospective cardiovascular risk in the general population, in patients with RA, adding hsCRP to the Framingham Risk Score and QRISK2 equations did not improve reclassification [12,14,29]. Furthermore, GlycA, and not hsCRP, provided additional value beyond traditional risk factors in association with subclinical CVD in psoriasis patients (30). Therefore, to adequately assess CVD risk in patients with chronic inflammatory diseases, new diagnostic tests that capture the residual CVD risk are needed. GlycA may represent one of these new diagnostic tests that one can leverage to detect ongoing active inflammation in high-risk states; moreover it is now available for physician use.

### 1.2 GlycA is a biomarker of systemic inflammation and CVD risk

As an alternative to traditional inflammatory markers, contemporary biomarkers of systemic inflammation including nuclear magnetic resonance (NMR)-measured GlycA, have been explored. GlycA quantifies systemic inflammation by assessment of an array of acute phase proteins [[31], [32], [33], [34]]. During ongoing systemic inflammatory processes, these proteins undergo circulating concentration and glycosylation pattern changes, and these alterations are reflected in the GlycA NMR signal [[31], [32], [33], [34]]. Theoretically, the GlycA NMR signal arises from all circulating glycosylated proteins in the liver. The proteins circulating at high enough concentrations to make significant contributions to the GlycA signal are the late acute phase reactants α1-acid glycoprotein, haptoglobin, α1-antitrypsin, α1-antichymotrypsin, and transferrin. Moreover, two of the major protein contributors to the GlycA signal, α1-acid glycoprotein and haptoglobin, are synthesized in and secreted from neutrophil granules, suggesting that, besides the liver, neutrophils may be a relevant source of elevated GlycA [[31], [32], [33], [34]].

GlycA concentrations are greater with both acute illnesses [35] and chronic inflammatory diseases and may be a better reflection of a systemic acute phase response than any single glycoprotein component since it is a composite marker stable over time and thus, has lower intra-individual variability [30,32,[36], [37], [38], [39], [40]]. In addition to high levels of systemic inflammation, GlycA concentrations correlate with the indolent inflammation present in cardiometabolic risk factors, obesity, insulin resistance, and the metabolic syndrome [33,39]. Thus, in chronic inflammatory diseases with low grade persistent inflammation, GlycA may serve as a biomarker of both systemic disease activity as well as cardiometabolic risk [30,32,[36], [37], [38], [39], [40]].

GlycA concentrations are associated with both prevalent and incident CVD events independent of traditional risk factors [32] (Table 1). GlycA is associated with the presence and extent of coronary artery disease (CAD) and peripheral artery disease (PAD) [[41], [42], [43]], and in persons with clinical and subclinical CVD [44,45]. Independent of traditional CVD risk factors and clinical parameters, GlycA concentrations are associated with incident CVD events, as noted in the Women’s Health Study (WHS) [46], the Prevention of Renal and Vascular End-stage Disease (PREVEND) Study [47], the Multi-Ethnic Study of Atherosclerosis (MESA) [48], the Intermountain Health Collaborative Study [49], the Atherothrombosis Intervention in Metabolic Syndrome with Low HDL/High Triglycerides: Impact on Global Health Outcomes (AIM-HIGH) trial [50], the Dallas Heart Study [51], and the Justification for the Use of Statins in Prevention: an Intervention Trial Evaluating Rosuvastatin (JUPITER) [52]. Moreover, multiple studies including the CATHGEN (CATHeterization GENetics) cardiac catheterization biorepository have demonstrated that GlycA is associated with not only presence or extent of coronary artery disease and cardiovascular mortality but also with all-cause mortality and non-cardiovascular mortality even when accounted for traditional cardiovascular risk factors. Notably, these prospective associations are only modestly attenuated—if at all—by hsCRP, implying that GlycA and hsCRP may denote different components of chronic inflammatory processes.

**Table 1:** GlycA’s role as a biomarker in CVD risk as evidenced in recent studies.

CV Events	Study type	Population	Study outcome	Study findings, sample size	Comparison
Women’s Health Study Akinkuolie et al.	Prospective cohort study	27,491 healthy women (mean age 54.7±7.1) were followed for a median of 17.2 years during which 1648 CVD events took place	Baseline GlycA concentrations associated with incident CVD	n=27,491 GlycA HR across quartiles 1-4: 1.00, 1.10(.92-1.30), 1.34(1.13-1.58) and 1.64(1.39-1.93) respectively p<0.0001 Median GlycA concentration: 369umol/L (326-416)	All CVD studies found that subjects with higher levels of GlycA had higher risk of CVD
GlycA, a Pro-Inflammatory Glycoprotein Biomarker, and Incident Cardiovascular Disease: Relationship with C-Reactive Protein and Renal Function. Gruppen et al.	Prospective cohort study	4759 participants who had no history of cancer or CVD	Participants with greater GlycA have greater incident CVD risk	n=4759 CVD risk in highest GlycA quartile after clinical and lipid adjustment was 1.58(1.05-2.37) P=0.004	
Comparison of the Predictive Value of GlycA and Other Biomarkers of Inflammation for Total Death, Incident Cardiovascular Events, Noncardiovascular and Noncancer Inflammatory-Related Events, and Total Cancer Events. MESA study Duprez et al.	Prospective cohort study	6523 healthy participants without overt CVD from the Multi-Ethnic Study of Atherosclerosis	GlycA concentration predictive of total death, fatal and nonfatal CVD and total cancer	n=6523 p=0.009 Mean GlycA 380.7±61.1umol/L GlycA predictive of CHD n=922. p=0.009 In minimal model for outcome prediction of any CVD GlycA is 1.27(1.19-1.35)	
Intermountain Heart Collaborative Study Muhlestein et al.	Prospective cohort study	2996 patients who had a coronary angiography followed for 7.0±2.8 years	Baseline GlycA concentrations were both independent and additive risk markers for MACE, HF hospitalizations and death	GlycA’s highest quartile was associated with future MACE HR:1.43(1.22-1.69) p<.0001	
Relations of GlycA and Lipoprotein Particle Subspecies With Cardiovascular Events and Mortality: A Post Hoc Analysis of the AIM-HIGH Trial Otvos et al.	Double blind placebo-controlled trial	2754 patients with controlled LDL-C levels who were treated with extended-release niacin	Inflammation is associated with greater CVD and death risks	Baseline levels of GlycA associated with CVD events had a HR of 1.17 P<0.0001 All-cause mortality was associated with baseline GlycA with a HR of 1.46 P<0.0001	
JUPITER study Akinkuolie et al.	Double blind placebo-controlled trial	12527 participants with low LDL and hsCRP ≥2 mg/L	Greater GlycA is associated with greater CVD risk independent of traditional risk factors	n=12527 Baseline GlycA levels associated with increased CVD risk: HR 1.20 (1.08–1.34) p=0.0006	
CATHGEN McGarrah et al.	Retrospective observational study	7617 subjects in the CATHGEN cardiac catherization biorepository	Greater GlycA associated with both presence and extent of CAD and with cardiovascular mortality GlycA and HDL subclasses found to have opposing effects on mortality risk	n=7617 GlycA associated with CAD presence: OR 1.07 (1.02-1.13) p=0.01 GlycA associated with CAD extent: OR 1.08(1.03-1.12 p<.0001 GlycA associated with cardiovascular mortality: 1.37(1.30-1.45) p<0.0001	N/A
Increased glycoprotein acetylation is associated with high cardiac event rates: Analysis using coronary computed tomography angiography An et al.	Cohort Study	342 patients who had a CCTA with no prior known CAD	Greater GlycA associated with greater MACE and death risks	In the adjusted model, following MACE resulted Low GlycA: 1.0 Intermediate GlycA: 1.41 (0.98-1.93) High GlycA: 1.91 (1.34-2.78) p<0.001 In the adjusted model, following all-cause death resulted Low GlycA: 1.0 Intermediate GlycA: 2.22 (1.53-3.09) High GlycA: 3.65 (2.62-5.09) p<0.001	N/A

### 1.3 GlycA is a marker of disease activity and CVD risk in patients with chronic inflammatory disease

GlycA is associated with both disease activity and CVD in patients with RA, SLE and psoriasis [32] and captures active and ongoing systemic inflammation (Table 2). In RA, GlycA levels are increased and strongly associated with disease activity by RA severity scores. Furthermore, GlycA was associated with coronary calcium scores [36,39]. Additionally, in SLE, GlycA concentrations are higher, increase with disease activity as assessed by the SLEDAI, and associate with non-calcified coronary artery plaque burden by coronary CTA in SLE patients [37,38,53]. In psoriasis, GlycA concentrations are also elevated, strongly associated with cutaneous disease severity by PASI scores and associated with subclinical vascular diseases assessed by FDG PET/CT aortic vascular inflammation and CTA derived coronary artery disease burden [30]. Interestingly, in that study, GlycA concentrations captured sub-clinical vascular disease incrementally over hsCRP, and also decreased when skin disease was treated suggesting it tracked disease activity [30]. Thus, GlycA may be an effective clinical tool for assessing both disease activity and CVD risk in patients with chronic inflammatory diseases, even when patients are on treatment with anti-inflammatory therapies [36,54].

**Table 2:** GlycA’s Role as a Biomarker in inflammatory Disease as Evidenced in Recent Studies.

RA Studies	Study type	Population	Study outcome	Study findings, sample size		Comparison
Utility of a novel inflammatory marker, GlycA, for assessment of rheumatoid arthritis disease activity and coronary atherosclerosis Ormseth et al.	Cross-sectional study	Cohort consists of patients characterized for CV risk All patient’s 18+ and RA and control groups were matched for age, sex and race	GlycA may be useful in assessing RA disease activity GlycA in RA associated with coronary artery atherosclerosis	GlycA n=166	398 μmol/L (348 to 473 μmol/L)	All studies had elevated GlycA levels in RA Mean RA GlycA in Ormseth et al. and Bartlett et al. study: 375.4 μmol/L Mean Control GlycA in Ormseth et al. and Bartlett et al. study: 336.4 μmol/L
				Control n=90	(344 μmol/L (314 to 403 μmol/L)	
				P<.001		
A novel biomarker, GlycA associates with disease activity in rheumatoid arthritis and cardio-metabolic risk in BMI-matched controls Bartlett et al.	Cross-sectional study	Participants were patients with RA versus controls that were sex, race and BMI matched Patients with known diabetes and CVD were excluded	GlycA associated with traditional inflammatory markers and cardio-metabolic sources in both RA and controls. Associations were stronger for traditional inflammatory markers in persons with RA and cardio-metabolic factors in those without RA	GlycA n=50	352.8 ± 67.2 μmol/L	
				Control n=39	328.9 ± 53.5 μmol/L	
				P=.036		
Characterization of ^1^H NMR Plasma Glycoproteins as a New Strategy To Identify Inflammatory Patterns in Rheumatoid Arthritis Fuertes-Martín et al.	Cross-sectional study	210 patients with RA versus 203 healthy control had GlycA levels measured with ^1^H NMR	Both GlycA and GlycB associated with inflammation in patients with high RA disease activity	When compared to the control group, RA patients showed a 10.65% increase in the GlycA associated areas *p* = 2.21 × 10^–10^		
**SLE Studies**	**Study type**	**Population**	**Study outcome**	**Study findings and sample size**		**Comparison**
GlycA, a Novel Marker of Inflammation, is Elevated in Systemic Lupus Erythematosus Chung et al.	Cross-sectional study	Patients with SLE were compared to controls and matched for age, sex and race	GlycA concentrations are greater in SLE patients GlycA is associated with inflammation markers	GlycA n=116	398 (350-445) μmol/L	All four SLE studies showed increase in GlycA concentrations in SLE patients. Mean SLE GlycA for Chung et al and Purmalek et al. 401.5 μmol/L Mean SLE control for Chung et al and Purmalek et al. 348.25 μmol/L
				Control n=84	339 (299-391) μmol/L	
				P<.001		
Longitudinal Evaluation of Lipoprotein Parameters in Systemic Lupus Erythematosus Durcan et al.	Longitudinal study	52 Patients in the Hopkins Lupus Cohort had their Sera collected and analyzed for lipoprotein and GlycA levels over 229 visits	GlycA greater in SLE than controls and associated with disease activity in SLE	In Univariate relationship in lipoprotein subtypes and clinical characteristics in the SLE disease activity index, GlycA had a clinically significant mean change		
				GlycA n=52	4.15	
				P=.0047		
Lipoprotein subfractions and glycoprotein acetylation with coronary plaque burden in SLE Purmalek et al.	Cross-sectional study	64 SLE patients (36 had CCTA) and 30 controls (18 had CCTA) Patients with GFR<60 mL/min were excluded	In SLE, GlycA and lipoprotein profiles associated with CVD risk Proatherogenic lipoprotein profile associated with premature CVD in patients with SLE	GlycA n =64	405.0 (365–470) μmol/L	
				Control n=30	357.5 (301–411) μmol/L	
				P<0.001		
**Psoriasis Study**	**Study type**	**Population**	**Study outcome**	**Study findings and sample size**		**Comparison**
GlycA Is a Novel Biomarker of Inflammation and Subclinical Cardiovascular Disease in Psoriasis Joshi et al.	Two stage cross sectional study with PENN and NIH cohorts	PENN cohort n=231 with psoriasis patients and controls NIH cohort n=181 with psoriasis and controls	GlycA more strongly associated than hsCRP with disease severity and subclinical CVD in psoriasis Treatment in psoriasis associated with reduction in GlycA concentrations.	PENN		Both the PENN and NIH trials showed increase in GlycA levels with Psoriasis Mean PSO GlycA: 412.3 μmol/L Mean control GlycA: 317.8 μmol/L
				GlycA n=122	408.8± 75.4 μmol/L	
				Control n=109	289.4± 60.2 μmol/L	
				P<.001		

				NIH		
				GlycA n=151	415.8± 63.2 μmol/L	
				Control n=30	346.2± 46 μmol/L	
				P<.001		

### 1.4 GlycA levels change in response to exercise, weight loss, and anti-inflammatory agents

Circulating GlycA levels are favorably modified by lifestyle changes such as exercise in the absence of dietary modifications [[55], [56], [57]]. Exercise training reduces GlycA independent of age, sex, race, baseline body mass index and beyond baseline GlycA levels, and occurred with 14 different interventions and seven distinct populations [56]. In addition to exercise training, GlycA is favorably modified by bariatric surgery with concomitant weight loss suggesting a tight association with body fat [58]. In persons with RA and psoriasis, GlycA concentrations are reduced with anti-inflammatory treatments using inhibitors of JAK1/2, TNFα, IL-17A or IL-12/IL-23 [30,[59], [60], [61], [62]], whereas GlycA concentrations are largely unaffected by treatment with a statin or extended release niacin [50,52]. Thus, in persons with chronic inflammatory diseases, GlycA may be useful clinically for monitoring disease activity and/or CVD risk reductions following both pharmacologic and lifestyle interventions.

### 1.5 Clinical perspective

GlycA can be measured in the general population as an additional marker of CVD risk for primary or secondary prevention. GlycA is strongly associated with CVD risk in patients with chronic inflammatory illnesses, even beyond hsCRP. Moreover, a value of 400 ​μmol/L has been accepted as the cut-point for systemic inflammatory states. In this population, those who have higher values have been shown to have more CVD risk. As for the general population, we would expect the cut-point to be lower when it comes to risk stratification regarding secondary prevention, however studies focusing on concrete cut-points are lacking. Given the available data, it appears that GlycA levels less than 400 ​μmol/L are relate to low CVD risk in chronic inflammation in those without overt clinical CVD. Levels greater than or equal to 400 ​μmol/L are indicative of greater inflammation-related CVD risk. Potentially, GlycA could serve to provide CV stratification in patients with RA, PSO and SLE when the value is beyond 400. GlycA levels above 400 ​μmol/L may trigger more aggressive measures for treatment of CV risk factors, recommendation of physical activity, weight loss, and perhaps more aggressive treatment of the underlying inflammatory disease. Early observational studies have shown promise of GlycA association with CV risk in inflammatory states beyond hsCRP, and support the conduct of prospective, randomized studies using GlycA to identify patients at high-risk for CVD [[30], [31], [32], [33], [34]].

## 2 Conclusions

Traditional assessments of CVD risk including lipid panel assessment, Framingham risk score, and hsCRP do not effectively capture the higher risk of CVD in chronic inflammatory diseases. Prediction of the CVD risk in these diseases may be improved with measurement of GlycA, a composite measure of concentrations and glycosylation of acute phase proteins. In chronic inflammatory diseases, GlycA concentrations associate with both traditional CVD risk factors as well as disease activity. GlycA concentrations respond to nonpharmacologic interventions targeting CVD risk, including exercise and weight loss, as well as pharmacologic agents targeting inflammatory pathways. Thus, in chronic inflammatory diseases, GlycA offers a promising tool to monitor systemic inflammation and CVD risk.

## Funding sources

This study was supported by the National Heart, Lung and Blood Institute (10.13039/100000050NHLBI) Intramural Research Program (HL006193- 06). The funding sources had no role in the design and conduct of the study; collection, management, analysis, and interpretation of the data; preparation, review, or approval of the manuscript; and decision to submit the manuscript for publication.

## Disclosures

Dr. Mehta is a full-time US government employee and has served as a consultant for Amgen, Eli Lilly, and Leo Pharma receiving grants/other payments; as a principal investigator and/or investigator for 10.13039/100006483AbbVie, 10.13039/100006436Celgene, 10.13039/100008897Janssen Pharmaceuticals, Inc, and 10.13039/100004336Novartis receiving grants and/or research funding; and as a principal investigator for the National Institute of Health receiving grants and/or research funding.

## Declaration of competing interest

The authors declare that they have no known competing financial interests or personal relationships that could have appeared to influence the work reported in this paper.
